# Corneal Debridement Combined with Intrastromal Voriconazole for Recalcitrant Fungal Keratitis

**DOI:** 10.1155/2018/1875627

**Published:** 2018-02-19

**Authors:** Yajie Sun, Zhuo Sun, Yukai Chen, Guohua Deng

**Affiliations:** The Third People's Hospital of Changzhou, Changzhou, China

## Abstract

**Background:**

To analyze the therapeutic effects of corneal debridement combined with intrastromal voriconazole in recalcitrant fungal keratitis.

**Methods:**

This is a retrospective study. Fourteen patients with recalcitrant fungal keratitis were treated by corneal debridement combined with intrastromal voriconazole (50 *μ*g/0.1 ml). This paper reviews and analyzes the patients' basic state, surgical intervention, medicinal treatment, and outcomes.

**Results:**

The mean sizes of infiltration and ulcer were (5.54 ± 1.32)mm and (3.46 ± 1.03)mm, respectively, and the mean depth was (315.43 ± 57.72)*μ*m. Twelve of the patients had satellite lesion, and 2 suffered hypopyon. After intrastromal voriconazole, the size of infiltration decreased significantly to (4.32 ± 1.10)mm (*P* < 0.001), but there was no significant change in ulcer size ((3.36 ± 0.92)mm, *P* = 0.082). Thirteen patients were cured after corneal debridement. The mean healing time was (15.38 ± 7.38) days. Excluding one cured patient with optic nerve atrophy and one patient for whom the treatment failed, the mean best-corrected visual acuity after healing was (0.23 ± 0.18)LogMAR, a significant improvement compared to pretreatment (0.87 ± 0.57(LogMAR), *P* = 0.01). The mean corneal astigmatism was (1.3 ± 1.6)D of 12 cured patients after healing and (1.0 ± 0.7)D at final follow-up, and there was no significant difference (*P* = 0.374).

**Conclusions:**

Corneal debridement combined with intrastromal voriconazole is a secure and effective treatment for recalcitrant fungal keratitis.

## 1. Background

Fungal keratitis is a serious cause of blindness worldwide, especially in developing countries. The risk factors are vegetative trauma, contact lens use and contact lens solution, ocular surface disease, topical steroid or antibiotic use and immunosuppressive systemic states, and so forth [1, 2]. Fungal keratitis has a poor prognosis because available antifungal drugs are limited by poor penetration, limited spectrum, and surface toxicity. In the past, therapeutic keratoplasty (TPK) was usually used to treat recalcitrant fungal keratitis, but there were some limitations, such as low success, severe complications, and decreased availability of donor corneas, especially in developing countries [3, 4]. Recent studies indicate that intrastromal injection of antifungal drugs could better control infection by increasing drug concentrations at the infection site [3, 5, 6]. Corneal debridement has been used for relatively superficial lesions to control infection by removing necrotic tissue containing toxic debris, pathogens, and inflammatory cells—products that could further damage corneal tissue—and improving the penetration of topical medicine [7]. Up to now, rare studies have been conducted on the approach of intrastromal voriconazole combined with corneal debridement [8]. The current study shows a good clinical effect by presenting data on 14 patients with recalcitrant fungal keratitis treated with intrastromal voriconazole combined with corneal debridement.

## 2. Methods

### 2.1. Materials

**Figure 1:**
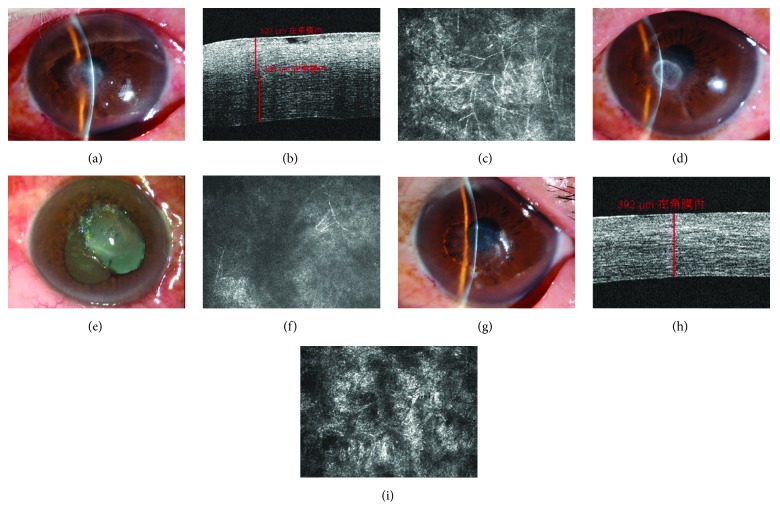
(a) Fungal ulcer located at the corneal center. The diameter of the ulcer was 5 mm, and it involved midstroma by slit lamp examination. (b) The depth of the ulcer was 320 *μ*m by AS-OCT. (c) Mass hyphae were found by ICVM before treatment. (d) After therapy of topical antifungal and intrastromal voriconazole (50 *μ*g/0.1 ml), the size of infiltration decreased compared with (a), but the ulcer was still obvious. (e) During debridement, the infiltrate and necrotic tissue were removed thoroughly. (f) Trifle hyphae were found by ICVM at 7 days after corneal debridement. (g) The corneal epithelial healed with a little nebula at 14 days after corneal debridement. (h) The residual depth of the cornea was 392 *μ*m by AS-OCT at 14 days after corneal debridement. (i) Corneal scar formed and stroma cell activated with no hyphae by ICVM at 14 days after corneal debridement.

**Figure 2:**
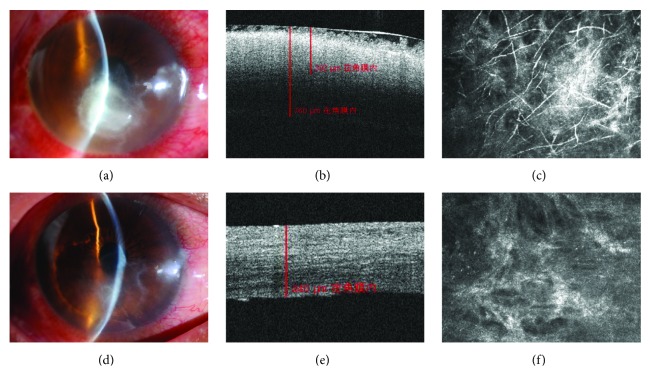
(a) The fungal ulcer (size: 5 mm) located in the paracentral of the cornea. This case suffered hypopyon of about 1 mm. (b) AS-OCT shows the strong reflection of the infiltrate; the depth was about 392 *μ*m. (c) Mass hyphae were found by ICVM pretreatment. (d) The corneal epithelium nearly healed with a little nebula at 6 days after corneal debridement. (e) AS-OCT showed the residual depth of cornea (340 *μ*m) at 8 days after corneal debridement. (f) No hypha was found by ICVM at 8 days after corneal debridement.

This retrospective study followed the tenets of the Declaration of Helsinki and was approved by the ethics committee of The Third People's Hospital of Changzhou. Written informed consent was obtained from each participant. All patients with fungal keratitis involving midstroma, not responding to topical antifungal medications including natamycin and voriconazole, were enrolled in the study. The diagnosis of fungal keratitis was based on positive results of in vivo confocal microscopy (IVCM) (Figures 1(c) and 2(c)) and mycological diagnosis. Cases with involvement of adjacent sclera, impending or frank corneal perforation, and concomitant endophthalmitis were excluded from the study. AS-OCT was performed to record the depth of lesion pretreatment and residual corneal depth after ulcer healing, respectively (Figures 1(b) and 2(b)). The size, location, and complications such as endothelial plaque, hypopyon, and satellite lesion were examined by slit lamp biomicroscopy. IVCM was performed postoperation to conform the residual hypha (Figures 1(f), 1(h), and 2(f)). BCVA was measured and recorded pretreatment, after epithelia healed and at final follow-up. Corneal astigmatism also was recorded after epithelia healed and at final follow-up.

### 2.2. Mycological Diagnosis and Antifungal Susceptibility Test

The material thus obtained from scraping was used for direct microscopic examination using Gram's stain and 10% KOH mount and also inoculated into Sabouraud's dextrose agar, blood agar, and chocolate agar for culture and identification of species by standard microbiological procedures [9]. Antifungal susceptibility testing was performed with AST YS07 kits on VITEK 2 compact. Standard operative procedures as described by the manufacturer were followed. Antifungal susceptibility testing was performed by NCCLS M44-A2 disc diffusion method [10].

#### 2.2.1. Medicine Preparation

Topical (1%) and intrastromal voriconazole (50 ug/0.1 ml) were prepared in the hospital pharmacy. The voriconazole powder of 200 mg (VFEN, Pfizer, USA) was reconstituted with 20 ml of lactated Ringer's solution (LR) to obtain 20 ml of clear concentrate containing 10 mg/ml of voriconazole. A 1 ml aliquot of this solution was further diluted with 19 ml of LR to a concentration of 0.5 mg/ml (50 ug/0.1 ml) for injection [11]. The voriconazole injection was prepared fresh each time, and the topical voriconazole was stored under aseptic conditions at a temperature of 2–8°C for 1 week [5, 11].

#### 2.2.2. Therapeutic Regimen

Topical natamycin sulfate (5%, Alcon) and voriconazole (1%, VFEN, Pfizer) were instilled every hour once fungal keratitis was conformed. The response to therapy was noted on slit lamp examination and defined as “no response to therapy” if there was no change in the size or depth of the ulcer or infiltrates and defined as “worsened” if there was an increase in size or depth of the ulcer or infiltrates, or perforation. If there was no response to the combined therapy for 2 weeks, intrastromal voriconazole (50 ug/0.1 ml) was performed around the lesion. In case of worsening or no response to the previous injection, intrastromal injection was repeated at an interval of 72 h. Corneal debridement was performed when gray infiltration and necrotic tissue were obvious even after the satellite lesion disappeared and the ulcer size diminished.

#### 2.2.3. Intrastromal Injection and Corneal Debridement

All intrastromal injections were performed under topical anesthesia (0.4% oxybuprocaine hydrochloride eye drops, Santen, Japan) in aseptic conditions using an operation microscope. The reconstituted voriconazole (50 ug/0.1 ml) was loaded in a 1 ml tuberculin syringe with a 30-gauge needle. With the bevel down, the needle was inserted obliquely in the uninvolved, clear area of the stroma to reach the infiltrate at the midstromal level. Voriconazole (0.05 ml) was injected in four divided doses around the infiltrate to form a drug deposit around the circumference of the lesion [5]. Circumferential injection ensured formation of a barrage of intrastromal voriconazole around the entire infiltrate.

Corneal debridement was also performed under topical anesthesia (0.4% oxybuprocaine hydrochloride eye drops, Santen, Japan) in aseptic conditions using an operation microscope. The corneal lesion was removed layer by layer, using a sterile crescent knife (Mani, Japan) and 0.12-microtoothed forceps, until the residual cornea was smooth with no obvious infiltrate (Figure 1(e)). The corneal tissue surrounding the lesion was then cut smooth with a micro scissor to facilitate corneal epithelial healing.

After intrastromal injection and corneal debridement, the patients were continued on the previously mentioned topical antifungal regimen. Patients were examined daily, and the response to therapy was recorded on the slit lamp. The infection was considered resolved when there was complete healing of epithelial defect with resolution of corneal infiltrate and no hyphae found by IVCM. Topical antifungal therapy was continued for at least 2 weeks after the complete resolution of infection. Patients with impending perforation and progressive worsening of infiltrates were taken up for keratoplasty.

### 2.3. Statistical Analysis

The data were analyzed using SPSS 19.0 statistical software. The continuous variables were presented as mean ± standard deviation, and the pair *t-*test was used to compare differences between pre and posttreatment. A *P* value of less than 0.05 was considered statistically significant.

## 3. Results

The details of fourteen patients (8 males, 6 females) enrolled in the study are shown in Table 1. The mean age of the patients was (53.6 ± 12.3) years (29~71 years). Except for one patient with optic nerve atrophy, the other 13 patients had no ocular or systemic complications. Hyphae were confirmed in all patients by IVCM and corneal smears. The organisms isolated were *Fusarium* species in 11 eyes and *Alternaria* species in 3 eyes. The organisms were resistant to voriconazole in 3 patients, intermediary sensitive to voriconazole in 2 patients, and sensitive to voriconazole in 8 patients. The mean sizes of infiltration and ulcer measured along the longest axis were (5.54 ± 1.32)mm (3.5~8 mm) and (3.46 ± 1.03)mm (2.0~5.5 mm), respectively, and the mean depth was (315.43 ± 57.72)*μ*m (188~392 *μ*m) by AS-OCT. Twelve of the patients had satellite lesion, and 2 suffered hypopyon. One patient had central lesion, 9 patients had paracentral lesion, and 4 patients had peripheral lesion. Excluding one patient with optic nerve atrophy, the mean BCVA for the other 13 patients was (0.87 ± 0.57)LogMAR.

Of the 14 patients enrolled in the study, the satellite lesion in 12 patients and hypopyon in 2 patients disappeared after intrastromal voriconazole, without secondary infection or corneal perforation. After injection, the size of infiltration decreased significantly to (4.32 ± 1.10)mm (*P* < 0.001), but there was no significant change in ulcer size (3.36 ± 0.92 mm, *P* = 0.082). All patients received corneal debridement after intrastromal voriconazole, and 13 of 14 patients were cured after corneal debridement. Of the 13 successfully treated patients, 7 received a single injection and 6 received two injections. The failure received 3 injections prior to corneal debridement. The mean number of injection in 13 cured patients was (1.46 ± 052). There was neovascularization in 3 patients with peripheral lesion (75%) and in 3 patients with paracentral lesion (37.5%) after treatment. The combined treatment failed in 1 patient who finally received therapeutic lamellar keratoplasty (LKP). The organism identified was *Fusarium* and was resistant to voriconazole in vitro.

The mean follow-up time was (37.14 ± 7.38) days, and healing time was (15.38 ± 7.38) days. The residue corneal depth after healing was (397.31 ± 65.55)*μ*m (324 *μ*m~500 *μ*m). Excluding the one cured patient with optic nerve atrophy and the patient for whom treatment failed, the mean BCVA was (0.23 ± 0.18)LogMAR after healing and (0.15 ± 0.08)LogMAR at final follow-up. The BCVA after healing showed significant improvement compared to pretreatment (*P* = 0.01), and the BCVA at final follow-up was better than that after healing (*P* = 0.023). The mean astigmatism was (1.3 ± 1.6)D of 12 cured patients after healing and (1.0 ± 0.7)D at final follow-up, and there was no significant difference (*P* = 0.374).

## 4. Discussion

Fungal keratitis is a vision-threatening infectious disease. It is managed mainly by antifungal agents. Keratoplasty or corneal transplant is usually reserved for acute management of corneal perforation and for visual rehabilitation following corneal scarring [8, 12]. Current antifungal agents are divided into four groups: polyenes, imidazoles, triazoles, and fluorinated pyrimidines [4]. These drugs can be administered topically, intravenously, or orally. However, they are limited by poor penetration, limited spectrum, ocular surface toxicity, limited clinical response, and prolonged course of treatment [4, 13, 14]. Natamycin, the only commercially available antifungal agent for ophthalmic use, has poor corneal penetration and precipitates on the corneal surface. Voriconazole has been reported to have a broad spectrum of antifungal properties and has been shown to be effective on ophthalmic clinical isolates, including the *Fusarium* and *Aspergillus* species [15, 16]. In vitro susceptibility data show that voriconazole has the best efficacy against pathogenic fungi compared with other agents [15, 17, 18]. Due to their poor ocular penetration, the effectiveness of antifungal drugs is limited when used to treat recalcitrant deep fungal keratitis. Thus, targeted treatment for fungal keratitis is key to managing the disease.

Intrastromal voriconazole has the potential to achieve adequate drug concentration at the site of infection through a targeted drug delivery process [3, 5, 11, 19]. An appropriate concentration of the drug was injected around the abscess, forming an adequate and persistent deposit around the circumference of the lesion to inhibit hyphae spread to the normal cornea [11]. In addition, since corneal stroma is stacked by regularly arranged lamellar without cross-linking between layers, the drug can diffuse in interlamination and form an adequate concentration at the bottom of the lesion. So far, some reports have shown a curative effect of intrastromal injection of 0.05% voriconazole, but the mean duration for healing was long and cure rates were various. Sharma et al. reported that 12 patients with fungal keratitis not responding to topical natamycin and voriconazole were treated with additional intrastromal voriconazole, and the mean healing time of 10 healed eyes was (39.75 ± 7.62) days [5]. Kalaiselvi et al. reported that 25 patients with recalcitrant fungal infection were treated with additional intrastromal voriconazole, and the mean resolution time of 17 healed eyes was (45.68 ± 11.49) days. In addition, they found that intrastromal voriconazole is a safe and effective way to treat deep recalcitrant fungal keratitis, and that *Fusarium* keratitis may show suboptimal response, but this needs further study [3]. Masanori et al. reported that intrastromal voriconazole injection is successful in treating yeast keratitis, but ineffective in treating filamentous fungal keratitis [20]. In a randomized clinical trial study, Sharma et al. compared the efficacy of topical voriconazole and topical natamycin with that of intrastromal voriconazole and topical natamycin in patients with recalcitrant fungal keratitis and concluded that intrastromal injections did not offer any beneficial effect over topical therapy [6].

In our research, 0.05% voriconazole was injected in four divided doses around the infiltration to ensure formation of a barrage of intrastromal voriconazole around the entire infiltration. The size of infiltration diminished significantly through the mean 1.46 injections of intrastromal voriconazole, but the ulcer did not shrink in size. Though the sensitivity of intrastromal voriconazole to different fungal genus remains to be determined, we can conclude that intrastromal voriconazole as an adjunctive therapy may be undertaken in selected patients who are unresponsive to other forms of antifungal therapy.

The same concentration of intrastromal voriconazole and the need for repeat injections to attain good results have been reported in many studies [3, 5, 8, 21]. Guber et al. reported that 3 patients with recalcitrant fungal keratitis were successfully treated by repeated intrastromal injection of voriconazole (100 mg/1 ml) in combination with corneal debridement and sustained no relative cornea damage [8]. However, recent studies have demonstrated that intracameral injection of ≥0.25% voriconazole could result in microstructural damage to corneal endothelial cells [22, 23]. Though the concentration of 0.05% intrastromal voriconazole has been reported to be effective in treating recalcitrant fungal keratitis [3, 5], further experiments relevant to intrastromal voriconazole injection are necessary to determine the optimal concentration of voriconazole with minimal toxicity to corneal endothelial cells.

In order to reduce the need for repeat injections and to accelerate corneal ulcer healing, corneal debridement was performed when the satellite lesion disappeared and the size of infiltration diminished (Figures 1(d) versus 1(a)) in our study. Corneal debridement could remove the dense hypha and necrotic tissue that could further damage healthy corneal tissue. In addition, corneal debridement could accelerate the penetration of topical antifungal drugs without the block of dense hypha and necrotic tissue. During the operation, after the removal of superficial gray coarse infiltrate, further debridement was done if stromal infiltrate and necrotic tissue were obvious and, after thorough debridement, there was no obvious infiltrate surrounding or at the bottom of the lesion. With this treatment, 92.9% (13 of 14) patients were cured without any complications (keratectasia or perforation), and the mean duration for healing was (12.8 ± 5.4) days. This period was significantly shorter than healing time in previous studies with intrastromal voriconazole alone. Kalaiselvi et al. reported success rates of 72% and 83% in treatments with intrastromal voriconazole and topical natamycin and voriconazole, respectively. In this study, however, a success rate of 92.9% was achieved in patients treated with intrastromal voriconazole and topical natamycin and voriconazole combined with debridement. The shorter treatment course and higher success rate could reduce associated economic pressure and mental stress.

The debridement procedure could cause corneal lamellar rupture and a decrease in corneal biomechanical function. Moreover, this may lead to iatrogenic keratectasia or corneal melting. The deeper and larger of debridement, the greater the loss of corneal function, and the higher the risk of complications. The key of success is to select suitable candidates. In our research, we estimated the depth of infiltrate and hyphae involved by AS-OCT and IVCM prior to debridement to determine whether debridement was suitable and the optimal depth of debridement. In addition, the organism-cultured outcomes were aided in determining whether debridement was feasible. We deemed that debridement is more effective in treating lesions with horizontal growth direction, such as *Fusarium* [24]. In this study, the locations of lesion were almost at the midperiphery of the cornea (13/14), the ulcers' size ranged from 2 mm to 5 mm, and the mean depth was 315.43 ± 57.72 *μ*m. The mean residual depth was 397.31 ± 65.55 *μ*m, a safe depth for the tension of intraocular pressure. While there was no keratectasia or corneal perforation in our study, 75% (3/4) of the patients with peripheral ulcer suffered corneal vascularization, and 37.5% (3/8) of those with paracentral ulcer suffered corneal vascularization.

In the available literature regarding intrastromal voriconazole for fungal keratitis, the BCVA improved differently [3, 5, 11]. Sharma et al. reported that patients in the intrastromal group had significantly worse BCVA after treatment compared with the topical group. The high proportion (90%) of central ulcers involving the visual axis in the intrastromal group (compared with 70% in the topical group) may have affected the outcomes in terms of BCVA after treatment [6]. In our study, the mean BCVA after epithelia healed improved significantly (*P* = 0.001), except in one case with optic atrophy and one in which LKP was performed, and the final BCVA improved further, accompanied by the fading of stromal edema (*P* = 0.023). Corneal debridement may affect BCVA by inducing irregular astigmatism, especially close to the optical zone, but we have no idea about the difference between intrastromal voriconazole and intrastromal voriconazole combined with debridement. A control study with a large sample should be necessary.

## 5. Conclusion

Intrastromal voriconazole combined with cornea debridement as adjunct to topical drugs is secure and effective for treating recalcitrant fungal keratitis. Suitable candidate screening and a thorough estimate before treatment are important for success.

## Figures and Tables

**Table 1:** Presentation and final outcome of cases with recalcitrant fungal keratitis that received intrastromal voriconazole combined with debridement.

Number	Size of infiltrate (mm)	Size of ulcer (mm)	Location	Depth (*μ*m)	Initial BCVA	Organism isolated	Sensitivity of Vori	Inhibition zone (mm)	Intervention	Residual depth (*μ*m)	Duration for healing (d)	NV	Final BCVA	Astig (D)
1	5.0	3.0	Peripheral	392	20/100	Fusarium	R	0	INJ = 1	324	9	Y	20/25	1.0
2	4.5	3.0	Paracentral	280	20/160	Fusarium	I	12	INJ = 1	408	13	N	20/25	1.75
3	5.5	4.0	Central	320	20/200	Fusarium	S	28	INJ = 2	392	20	N	20/32	1.0
4	6.0	4.0	Paracentral	332	20/63	Fusarium	S	22	INJ = 2	365	22	N	20/20	0
5	7.0	4.0	Paracentral	350	LP	Fusarium	R	10	INJ = 2	500	24	Y	LP	—
6	3.5	2.0	Peripheral	188	20/40	Alternaria	S	21	INJ = 1	476	10	N	20/25	0
7	8.0	5.0	Paracentral	392	20/2000	Fusarium	S	18	INJ = 2	340	14	Y	20/50	1.5
8	5.0	3.0	Peripheral	270	20/2000	Fusarium	S	18	INJ = 1	456	11	Y	20/32	2.0
9	4.0	2.0	Peripheral	236	20/50	Alternaria	S	21	INJ = 1	500	7	Y	20/25	0.5
10	6.5	4.0	Paracentral	344	20/80	Fusarium	S	23	INJ = 2	383	28	N	20/32	0
11	5.5	3.5	Paracentral	348	20/160	Fusarium	R	0	INJ = 1	329	9	N	20/32	2.0
12	4.5	2.5	Paracentral	300	20/63	Fusarium	I	12	INJ = 1	358	8	N	20/25	1.0
13	7.5	5.5	Paracentral	360	20/63	Alternaria	S	15	INJ = 2	334	25	Y	20/25	1.0
14	5.0	3.0	Paracentral	304	20/100	Fusarium	R	0	INJ = 3, LKP	—	32	—	20/25	3.0

BCVA: best-corrected visual acuity; LP: light projection; Vori: voriconazole; R: resistance; I: intermediary; S: sensitive; INJ: number of intrastromal voriconazole injection; NV: neovascularization; Astig: astigmatism.

## Data Availability

The datasets analyzed during the current study are available from the corresponding author on reasonable request (Email: czdgh1975@sina.com).

## Abbreviations

AS-OCT: Anterior segment optical coherence tomography
IVCM: Laser scanning *in vivo* confocal microscopy
BCVA: Best-corrected visual acuity
LKP: Lamellar keratoplasty.
